# Effects of Soybean- and Cottonseed-Based Diets on Growth Performance and Gut Microbiota of Black Soldier Fly Larvae

**DOI:** 10.3390/insects17070675

**Published:** 2026-06-28

**Authors:** Xingbao Feng, Ying Cui, Song Liu, Qianru Mo, Jingyi Hu, Xinyu Duan, Jiaxuan Jian, Ling Tian, Huiyu Yi, Zhijun Huang

**Affiliations:** 1 College of Animal Science, South China Agricultural University, Guangzhou 510642, China; 2025116@hbmzu.edu.cn (X.F.); u2440295342@163.com (Y.C.); 20243139061@stu.scau.edu.cn (S.L.); moqianru@stu.scau.edu.cn (Q.M.); 13957766790@163.com (J.H.); 18841743561@163.com (X.D.); 13760632372@163.com (J.J.); tianling@scau.edu.cn (L.T.); 2 College of Biological and Food Engineering, Hubei Minzu University, Enshi 445000, China

**Keywords:** black soldier fly, gut microbiota, anti-nutritional factors, protein feed, metabolic pathways

## Abstract

The impacts of various feed substrates and processing treatments (boiling and extrusion) designed to reduce plant-derived antinutritional factors on the growth and development, nutritional composition, and intestinal microbiota of black soldier fly larvae (BSFL) were evaluated. The research revealed that, except for the soybean flour group, the cottonseed meal group and the treatment group with antinutritional factors removed showed better growth trends compared to the Gainesville House Fly Diet group. The heat-processed groups had higher maximum larval weights, survival rates, and feed conversion rates than the untreated groups. Additionally, BSFL fed with high-protein and high-fat experimental diets generally exhibited higher fat and protein contents. Plant-derived antinutritional factors in the test substrates significantly affected the intestinal microbiota and their metabolic pathways in BSFL. Gut microbiota—including phyla such as *Firmicutes*, *Proteobacteria*, and *Bacteroidetes*, along with various lactic acid—and short-chain fatty acid-producing genera—and metabolic pathways related to carbohydrates, amino acids, cofactors, and vitamins, play crucial roles in helping BSFL resist plant antinutritional factors.

## Simple Summary

Black soldier fly is an economically significant insect with high feed conversion efficiency. It can transform nutrients into various organic substrates into proteins and fats, which can be used in the formulation of feeds for a wide variety of livestock. However, different feed substrates can also influence the growth traits, nutritional composition, and intestinal microbiota of black soldier fly larvae (BSFL). In this study, soybean flour and cottonseed meal were used as the primary constituents of feed substrates for BSFL. Through formulation and eliminating antinutritional factors, the research aimed to produce BSFL with high protein and fat content and to investigate the effects of plant antinutritional factors on larval development and the role, if any, of gut microbiota in mitigating these factors. These studies provide a reference feed formulation for the production of high-protein, high-fat and edible BSFL, while offering reliable scientific evidence regarding the role of intestinal microbiota in helping BSFL to resist plant antinutritional factors.

## 1. Introduction

*Hermetia illucens*, commonly known as the black soldier fly (BSF), is native to the Americans and has been widely distributed across tropical, subtropical, and temperate regions through human activity [1]. As a holometabolous insect, the larvae are saprophagous, capable of converting various organic wastes into their own biomass [2,3,4]. On a dry weight basis, the black soldier fly larvae (BSFL) contain approximately 40% crude protein and 30% lipids [5] and have been approved as an animal feed ingredient used for poultry, livestock, reptiles, and aquatic organisms in many countries [6,7,8]. Additionally, insect fats and proteins can be extracted and utilized as feed additives or for formulating new feed products. The relatively high crude fat content of BSFL renders it a viable substrate for biodiesel production. Chitin, another significant component of BSF biomass, enables the extraction of chitin and chitosan from these insects, which find applications in biomedicine, environmental protection, and various other fields [9,10]. The excreta of BSF can be utilized as a fertilizer and also employed for biogas production [11]. As research on BSF delves deeper, this insect has emerged as an increasingly vital resource, garnering widespread attention from various sectors.

Although BSFL possess rapid and efficient conversion capabilities [7], the quality of their food sources can influence their growth, development, and conversion efficiency. For instance, high-fat feed can increase the fat content of the larvae, but it may suppress their growth performance [12]. In contrast, a higher carbohydrate content contributes to enhancing the overall growth efficiency of the larvae. The ratio of protein to carbohydrates in the food source significantly impacts the growth rate and body composition of the larvae. When fed a vegetable residue-based diet with a protein-to-carbohydrate ratio of 1:2 and a total protein and carbohydrate content of 47%, the larvae exhibit optimal performance [13]. Further research has indicated that protein is a crucial nutrient for the growth and development of BSFL. A high-protein diet substantially enhances the biomass growth and conversion efficiency of BSFL. For example, when larvae are fed protein-rich feed, their weight gain and protein deposition rates significantly increase [14,15]. Moreover, the protein content also affects the fat and carbohydrate metabolism of the larvae, thereby influencing their ultimate application value [14].

At present, the commonly used feed for rearing BSFL in laboratories is the Gainesville House Fly Diet (GHFD). Comprising 50% wheat bran, 30% alfalfa meal, and 20% corn meal, this diet can effectively meet the growth and developmental needs of BSF larvae [16]. However, based on its ingredient analysis, GHFD has a relatively low protein content and may not be the optimal feed for BSF larvae. Soybean flour (SBF) serves as the primary plant protein source in current feeds, offering balanced nutrition and an amino acid profile similar to that of animal protein. Cottonseed meal (CSM) is also a readily available protein source in China. Both SBF and CSM can be used as major protein sources to improve the BSF larvae feed. Nevertheless, soybeans contain antinutritional factors such as trypsin inhibitors, soybean agglutinin, and phytic acid [17,18,19], while CSM contains gossypol, cyclopropenoid fatty acids, phytic acid, tannins, and other antinutritional factors [20]. These factors not only reduce their nutritional value but may also adversely affect the growth of BSF larvae. Therefore, in this experiment, we modified the feed formulation by using SBF and CSM—as well as these two protein sources processed through heating and extrusion methods to remove antinutritional factors—mixed with corn flour and rice bran. The aim is to rear BSF larvae with high protein or high fat content, providing theoretical basis and practical guidance for the development of artificial feeds and high-quality BSFL. Additionally, by analyzing the intestinal metagenomes of BSF larvae fed with different feed ingredients and subjected to various processing methods, we explored the responses of intestinal microbiota and their metabolic pathways to different substrates and antinutritional factors. This approach offers insights from a microbial perspective into how BSF larvae resist plant antinutritional factors.

## 2. Materials and Methods

### 2.1. Preparation of Experimental Larvae

BSF were provided by Bioforte Biotechnology (Shenzhen) Co., Ltd. (Shenzhen, China) BSF eggs laid within a unified 12 h period were collected and placed in plastic boxes measuring 17 × 11 × 7 cm. These boxes were covered with nylon mesh to ensure proper ventilation. Subsequently, the boxes were placed in a constant-temperature incubator for hatching. The incubation temperature was set at 30 °C, and the environmental relative humidity was maintained at approximately 75%. The entire hatching period was kept under complete darkness. After hatching, the BSFL was reared on GHFD at 28 °C with an environmental relative humidity of 75% and a 12 h light/12 h dark photoperiod for 5 days prior to use in subsequent experiments.

### 2.2. Feed Ingredient Composition and Dietary Formulation

All experimental feed raw materials were purchased from official stores on the Taobao e-commerce platform, and the specific purchasing sources were specified as follows: SBF from Manquan Flagship Store (Shanghai, China); CSM, extruded soybean flour (ESBF) and wheat bran from Jiahui Feed Store (Hebei, China); alfalfa meal from Yinong Home Furnishing Specialty Store (Shenzhen, China); and rice bran was directly supplied by Lingfeng Feed Factory (Zhangzhou, China). Their conventional nutritional values were referred to and sorted out according to the Chinese Feed Composition and Nutritional Value Table (35th Edition), and the results were presented in Table 1 [21].

Six experimental diet groups were formulated, and the detailed formulations were presented in Table 2. All raw feed ingredients were crushed and sieved through a 1.0 mm standard test sieve to achieve a uniform particle size. They were then thoroughly mixed according to the dry weight proportions shown in Table 2, followed by the addition of appropriate amounts of water to adjust the moisture content to approximately 70%. For the boiled soybean flour (BSBF) and boiled cottonseed meal (BCSM) treatments, the corresponding proportions of raw SBF or CSM were weighed, mixed with an appropriate amount of water, and boiled separately for 20 min. The boiled materials were then combined with rice bran and corn flour, and the final moisture content was adjusted to approximately 70% by calculation. ESBF used in this experiment was a commercially available finished product; it only underwent crushing and sieving through a 1.0 mm sieve without any further processing.

Based on the conventional nutritional values of raw materials listed in Chinese Feed Composition and Nutritional Value Table (35th Edition) (Table 1) and experimental diet formulations (Table 2), the nutritional levels of each diet were calculated and sorted out, as presented in Table 3.

### 2.3. Growth and Developmental Traits

For each experimental group, one hundred 5-day-old BSFLs were placed in plastic containers containing 150 g (wet weight) of experimental feed. All experimental diets were prepared using dry raw materials. During the experiment, distilled water was regularly sprayed to uniformly adjust and maintain the final feed moisture content at approximately 70% across all groups, so as to ensure consistent rearing conditions. The containers were covered with nylon mesh to prevent the larvae from escaping. Subsequently, they were placed in a constant-temperature incubator set at 28 °C with 75% humidity for rearing. Three replicate containers were set up for each of the six feed treatments. Every two days, 10 larvae were randomly sampled from each container using a five-point sampling method as follows: one at the center and four at the corners of the container, with 2 larvae collected per point. Larvae were then weighed individually using an electronic analytical balance (JA3003, HengPing, Shanghai, China) and returned to their container. During each sampling for larval weight, the number of surviving larvae and number of prepupal larvae were also counted for each container. The trial was terminated when 50% of BSFL in each replicate entered the prepupal stage, and the residual feed weight of each replicate was then recorded. During the collection of residual feed, visible larval frass was manually removed in advance. The remaining pure feed was dried at 60 °C for 48 h and then weighed for calculation. The survival rate and conversion rate at the prepupal stage were then calculated using the following formulas:

survival rate% = (number of larvae at the end of experiment/number of larvae at the beginning of the experiment) × 100%

conversion rate = (larval weight at the ending of the experiment − larval weight at the beginning of the experiment)/(initial substrate weight − final substrate weight) × 100%

### 2.4. Measurement of Nutritional Components of BSFL

When half of the larvae reached the prepupal stage, the larvae were collected for nutritional component determination. The collected BSFLs were continuously dried at 55 °C for 3 days, and then relevant nutrient indices were determined. Crude protein content was measured via the Kjeldahl method according to GB/T 6432-2018 [22], while crude fat content was determined using the Soxhlet extraction method specified in GB/T 6433-2006 [23,24].

### 2.5. Collection of BSFL Digestive Tracts

BSFLs that had grown to day 13 were selected. After being subjected to a 24 h starvation treatment, they were thoroughly washed with tap water and set aside for use. The external surface of the larvae was cleaned with 75% alcohol. They were then soaked in sterile water for 5 min in a laminar flow hood. After that, the larvae were removed with forceps, rinsed with sterile water, and the moisture on their surface was blotted dry using absorbent paper. The abdomen of the larvae was gently incised with dissecting scissors, and the complete intestinal tract (including foregut, midgut and hindgut) was taken out with forceps. Surface moisture was removed with filter paper, and the samples were then placed into 1.5 mL centrifuge tubes and stored at −80 °C. For each replicate, 30 BSFLs were randomly selected for dissection to extract intestinal samples, ensuring that the total sample amount in each group was ≥200 mg.

### 2.6. Metagenomic Sequencing

Total microbial community DNA extraction was carried out using the DNeasy Power Water Kit from Mo Bio/QIAGEN (Hilden, Germany). Subsequently, the extracted DNA was detected. A fluorescence spectrophotometer (NanoDrop One Spectrophotometer, Thermo Fisher Scientific, Waltham, MA, USA, A30221) was employed to measure the absorbance values of DNA at 260 nm and 280 nm, respectively, for determining DNA concentration. Additionally, the quality of DNA was assessed using 1% agarose gel electrophoresis. The concentration of the DNA solution was adjusted. The working DNA solution was stored at 4 °C, while the stock solution was stored at −20 °C. For library construction and sequencing, the standard Illumina TruSeq DNA Library Preparation Protocol (Illumina TruSeq DNA Sample Preparation Guide) was adopted to construct the required genomic sequencing libraries.

For metagenomic data, raw reads were quality-filtered and trimmed to remove low-quality reads and adapters. Clean reads were assembled, and open reading frames were predicted and annotated against the NCBI nr/nt, eggNOG, and KEGG databases. Taxonomic composition, α-diversity, β-diversity, and differential abundance analysis were performed to characterize the gut microbial community. LEfSE analysis (LDA threshold = 3) was used to identify significantly different taxa and metabolic pathways between groups. All metagenomic analyses were performed using the online platform GenesCloud (https://www.genescloud.cn/home, accessed on 29 December 2025) provided by Shanghai Personal Biotechnology Co., Ltd. (Shanghai, China).

### 2.7. Statistical Analyses

All results were presented as the mean ± standard deviation, with a minimum of three biological replicates included. Data visualization and analysis were performed using GraphPad Prism 9 and Excel. The normality of each experimental dataset was evaluated through the Shapiro–Wilk test. Statistical comparisons were conducted using one-way analysis of variance (ANOVA), and the significance was determined by the Tukey post hoc test. The difference was considered statistically significant when *p* < 0.05.

## 3. Results

### 3.1. Larval Growth Characteristics and Nutritional Components Fed with Different Diet

**Figure 1:**
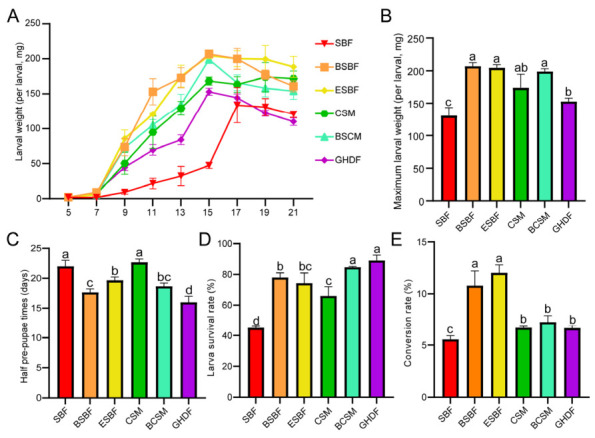
Growth, development and feed conversion rate of BSFL fed various feed formulations. (**A**) Larval weight; (**B**) maximum larval weight; (**C**) median time to the prepupal stage; (**D**) larval survival rate; (**E**) feed conversion rate. SBF, soybean flour-based diet; CSM, cottonseed meal-based diet; BSBF, boiled soybean flour-based feed; ESBF, extruded soybean flour-based feed; BCSM, boiled cottonseed meal-based feed; GHDF, Gainesville House Fly Diet.

With the exception of the SBF group, which exhibited slower growth compared to the GHDF group in the early stage (the first 12 days), all other groups demonstrated a better growth trend than the GHDF group throughout the entire growth cycle. Although the SBF group grew more slowly initially, it showed rapid growth from day 15 to day 17 and its weight surpassed that of the GHDF group after day 19 (Figure 1A). However, in terms of maximum larval weight, the SBF group recorded 133.67 ± 25.32 mg/larva, which was significantly lower than that of the GHDF group (153.00 ± 5.00 mg/larva). In contrast, the BSBF, ESBF, and BCSM groups had maximum larval weights of 207.00 ± 5.57, 204.67 ± 4.73, and 199.00 ± 4.00 mg/larva, respectively, all significantly higher than that of the GHDF group. The CSM group, with a maximum larval weight of 174.00 ± 20.88 mg/larva, showed no significant difference from the GHDF group. (Figure 1B, Table S1). Regarding the time for 50% of the BSFL in each group to reach the prepupal stage, the SBF group and CSM group had the longest time, followed by the BSBF group, ESBF group, and BCSM group, all of which were significantly longer than that of the GHDF group (Figure 1C). The larval survival rate in the BCSM group was comparable to that of the GHDF group and significantly surpassed the rates observed in the other groups. Notably, the SBF group exhibited the lowest larval survival rate among all. Moreover, the treated groups, namely BSBF, ESBF, and BCSM, demonstrated significantly higher larval survival rates compared to their corresponding untreated counterparts (Figure 1D). The conversion rates reflect the performance of BSFL in converting different feeds. The BSBF and ESBF groups exhibited the highest conversion rates, markedly outperforming not only the other experimental groups but also the GHDF group. Conversely, the SBF group registered the lowest conversion rate, falling significantly short of both the remaining groups and the GHDF group (Figure 1E). Nutritional analysis of the insect specimens demonstrated that, with the exception of the ESBF group, the crude protein content in the larvae of all other groups surpassed that of the GHDF group. As for the crude fat content within the larvae, every other group exhibited significantly elevated levels compared to the GHDF group (Table 4).

### 3.2. Quality Analysis of the Metagenomic Sequencing Data

The metagenomic sequencing results revealed that each sample generated between 5,404,589,584 and 7,773,428,962 raw bases, along with 35,791,984 to 51,479,662 reads. After quality filtering, a total of 6,070,713,807 to 6,342,611,796 clean bases and 35,292,566 to 50,723,336 clean reads were obtained. Among them, the proportion of valid sequence bases in each sample exceeded 98.23%, and the proportion of valid sequences was over 98.37%. Moreover, the Q30 base quality for each sample was greater than 96.24%, indicating high sequencing quality and reliability. The GC content ranged from 39.29% to 43.63%, and the goods coverage exceeded 99%, suggesting sufficient sequencing depth (Table S2).

### 3.3. Analysis of Intestinal Microbiota in BSFL Fed with Different Diet

Analysis of the metagenomic sequencing results of the intestinal microbiota of BSFL in the GHDF, SBF and CSM groups revealed that among the α-diversity indices, Simpson, Pielou’s evenness, and Shannon exhibited similar trends across the three groups. This indicates that microbial diversity and evenness are comparable (Figure S1A). In contrast, the Chao1 and observed species indices were higher in the GHDF group, suggesting differences in microbial abundance between the GHDF group and the other groups (Figure S1A). Principal Coordinate Analysis (PCoA) was employed to assess the similarity of microbial community structures (β-diversity) among the groups. The PCo1 value accounted for 61.70% of the variation, and the PCoA plot demonstrated a tendency for microbial communities to separate among the groups (Figure S1B).

**Figure 2:**
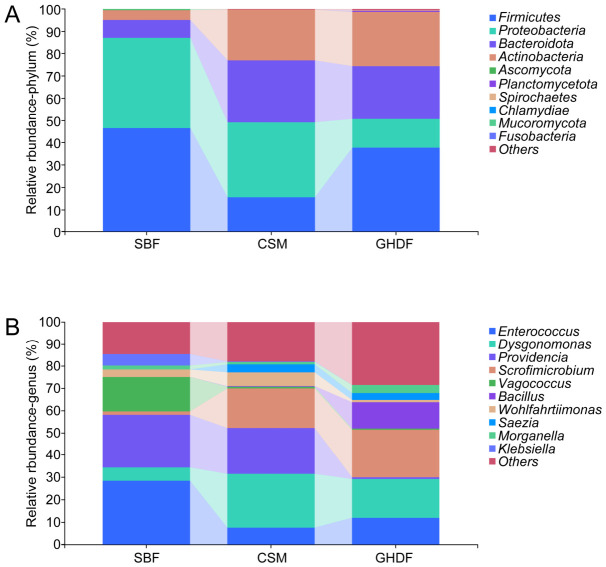
Gut bacterial composition of BSFL fed different diets. (**A**) Phylum-level composition; (**B**) genus-level composition. SBF, soybean flour-based diet; CSM, cottonseed meal-based diet; GHDF, Gainesville House Fly Diet.

**Figure 3:**
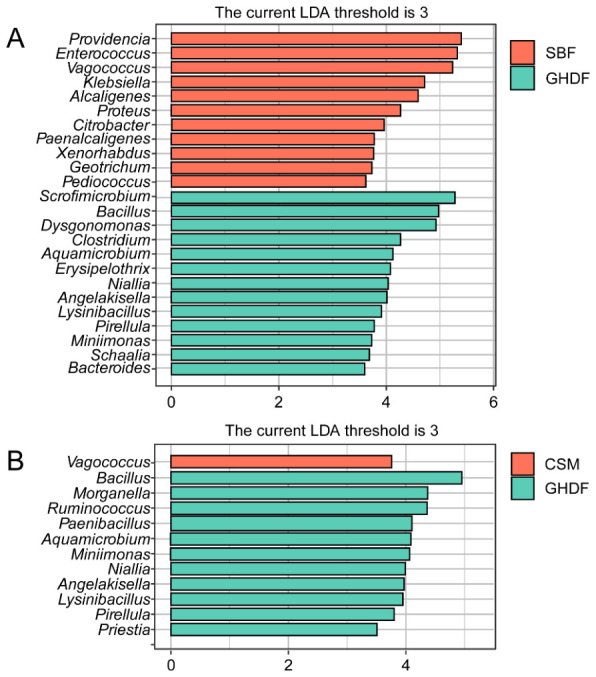
LEfSE analysis of genus-level gut microbiota differences in BSFL fed different diets. (**A**) SBF vs. GHDF; (**B**) CSM vs. GHDF. SBF, soybean flour-based diet; CSM, cottonseed meal-based diet; GHDF, Gainesville House Fly Diet.

Abundance analysis of intestinal microbiota at the phylum level across the three sample groups revealed that *Proteobacteria*, *Firmicutes*, *Bacteroidetes*, and *Actinobacteria* were the dominant phyla in all three groups, yet there were variations in their relative abundances among the groups. In the GHDF group, *Firmicutes* was the most abundant phylum (37.85%), followed by *Actinobacteria* (24.32%), *Bacteroidetes* (23.88%), and *Proteobacteria* (12.75%). *Firmicutes* (46.46%) and *Proteobacteria* (40.52%) were the dominant phyla in the SBF group, with *Bacteroidetes* (8.16%) and *Actinobacteria* (4.23%) ranking next. In the CSM group, *Proteobacteria* showed the highest abundance (33.67%), followed by *Bacteroidetes* (27.85%), *Actinobacteria* (22.67%), and *Firmicutes* (15.52%) (Figure 2A, Table S3). Venn diagrams were constructed to evaluate the operational taxonomic unit (OTU) distribution at the genus level among the three sample groups. The results indicated that there were 760 OTUs shared by all three groups. The number of OTUs unique to GHDF, SBF and CSM were 572, 61 and 91, respectively (Figure S2A). The results of the classification are as follows—composition Circos plot revealed that the top 10 bacterial genera in terms of abundance across the three groups were *Enterococcus*, *Providencia*, *Scrofimicrobium*, *Dysgonomonas*, *Vagococcus*, *Morganella*, *Bacillus*, *Wohlfahrtiimonas*, *Klebsiella* and *Saezia* (Figure S2B). Further analysis using classification—composition bar charts showed that the top five genera by abundance in the GHDF group were *Scrofimicrobium* (21.56%), *Dysgonomonas* (17.13%), *Enterococcus* (12.10%), *Bacillus* (12.03%) and *Morganella* (3.63%). In the SBF group, the most representative genera were *Enterococcus* (28.42%), *Providencia* (23.76%), *Vagococcus* (15.45%), *Dysgonomonas* (6.08%) and *Klebsiella* (5.03%). The five genera with the highest abundance in the CSM group were *Dysgonomonas* (23.90%), *Providencia* (20.65%), *Scrofimicrobium* (17.82%), *Enterococcus* (7.65%) and *Wohlfahrtiimonas* (6.20%) (Figure 2B, Table S4). LEfSE (with an LDA threshold of three) was employed to analyze the specificity of taxonomic units with significant differences among substrates in sample groupings. The results indicated that, compared to the GHDF group, the SBF group exhibited an increased relative abundance of 11 genera, including *Providencia*, *Enterococcus*, *Vagococcus*, *Klebsiella*, as well as *Alcaligenes* (Figure 3A). In contrast, in the CSM group, only the abundance of *Vagococcus* showed a significant increase (Figure 3B).

### 3.4. Differences in Metabolic Pathways of Intestinal Microbiota in BSFL Fed with Different Diet

**Figure 4:**
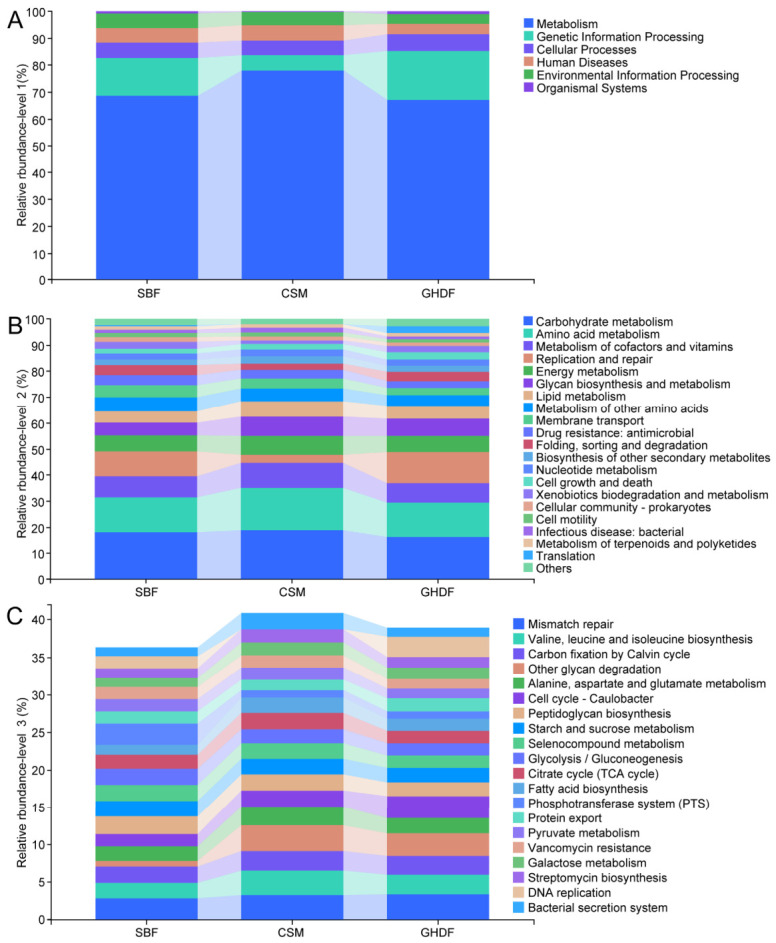
Metabolic pathway analysis of gut microbiota in BSFL fed different diets. (**A**) KEGG level 1; (**B**) KEGG level 2; (**C**) KEGG level 3. SBF, soybean flour-based diet; CSM, cottonseed meal-based diet; GHDF, Gainesville House Fly Diet.

**Figure 5:**
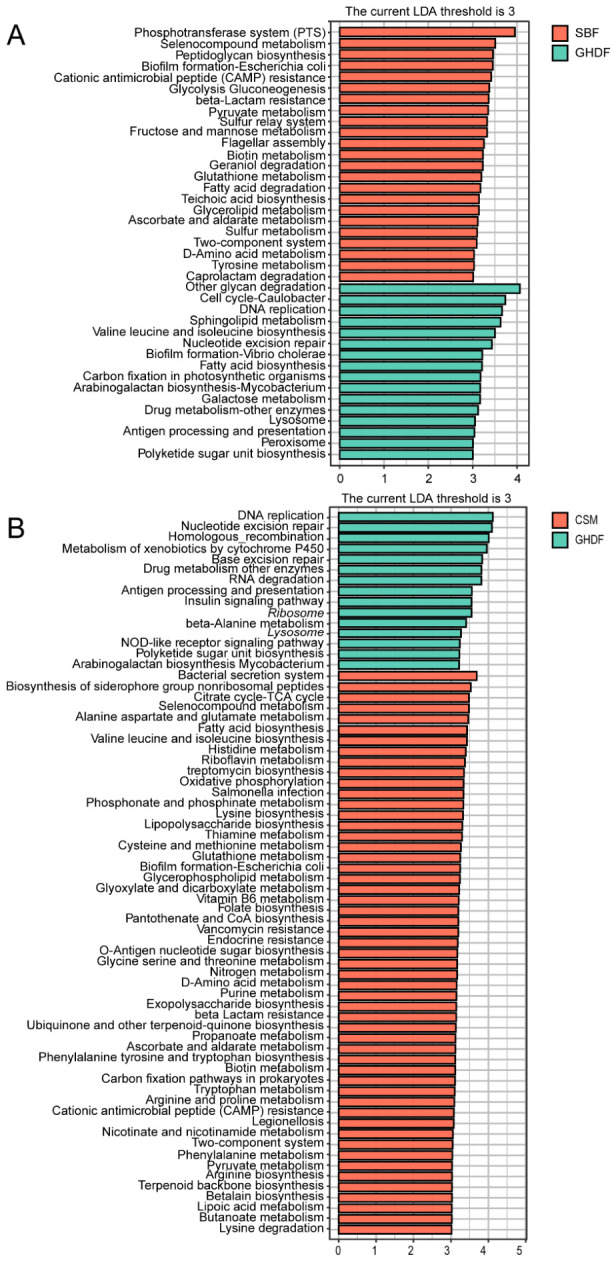
LEfSE analysis of differential metabolic pathways in BSFL fed different diets. (**A**) SBF vs. GHDF; (**B**) CSM vs. GHDF. SBF, soybean flour-based diet; CSM, cottonseed meal-based diet; GHDF, Gainesville House Fly Diet.

KEGG was utilized to evaluate the metabolic pathways of intestinal microbiota under different substrate conditions, namely SBF, CSM, and GHDF. The results revealed that at level 1, the predominant metabolic pathway across the three groups was metabolism, with its biological functional scope ranging from 67.30% to 78.16%. This was followed by metabolic processes such as Genetic Information Processing and Cellular Processes (Figure 4A). At level 2, the main enriched metabolic pathways in the three groups included carbohydrate metabolism, amino acid metabolism, metabolism of cofactors and vitamins, replication and repair and Energy metabolism (Figure 4B). At level 3, the metabolic pathways with relatively high abundances are Mismatch repair, Valine, leucine and isoleucine biosynthesis, Carbon fixation by the Calvin cycle, Other glycan degradation, and Alanine, aspartate and glutamate metabolism (Figure 4C). LEfSE (with an LDA threshold of three) was used to analyze the differences in level 3 metabolic pathways of intestinal microbiota across different substrate groups. The results demonstrated that, compared to the GHDF group, the upregulated metabolic pathways of SBF group included Phosphotransferase system (PTS), Selenocompound metabolism, Peptidoglycan biosynthesis, Biofilm formation—Escherichia coli, as well as Cationic antimicrobial peptide (CAMP) resistance, with a total of 23 pathways identified. Of these, four pathways were associated with carbohydrate metabolism and three with amino acid metabolism (Figure 5A). The CSM group showed upregulated metabolic pathways such as Bacterial secretion system, Biosynthesis of siderophore group nonribosomal peptides, Citrate cycle (TCA cycle), Selenocompound metabolism, and Alanine, aspartate, and glutamate metabolism, totaling 53 pathways. Among them, 16 pathways were related to amino acid metabolism, nine to the metabolism of cofactors and vitamins, and eight to carbohydrate metabolism (Figure 5B).

### 3.5. Differences in Intestinal Microbiota Under Different Substrate Treatment Methods

SBF contains numerous antinutritional factors, and different treatment methods have varying impacts on these factors. Therefore, we conducted metagenomic sequencing on SBF subjected to the following three distinct treatment methods: no treatment (SBF), Boiled treatment (BSBF), and Extruded treatment (ESBF), aiming to analyze the effects of different treatments on intestinal microbiota. Analysis of the sequencing results revealed that the α-diversity indices in all three groups exhibited similar trends, indicating that the microbial diversity, evenness, and abundance were comparable (Figure S3A). PCoA was used to assess the similarity of microbial community structures among the groups (β-diversity). The PCoA plot demonstrated a tendency for microbial communities to separate between the groups, with a PCo1 value of 43.7% (Figure S3B).

**Figure 6:**
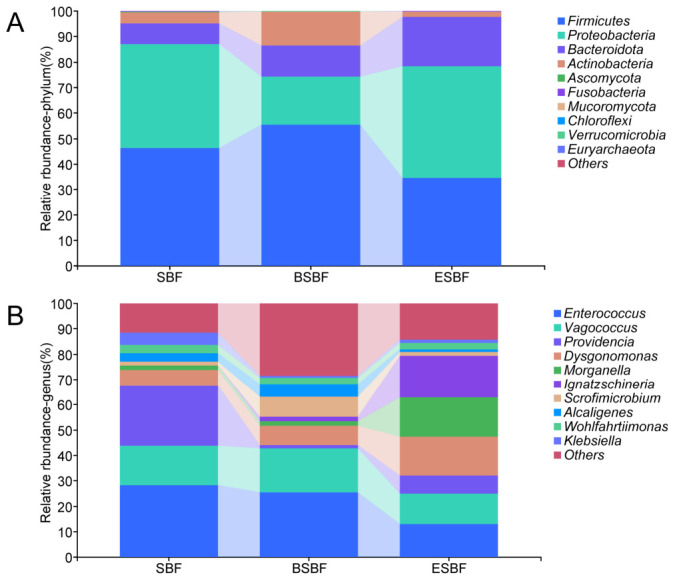
Gut bacterial composition of BSFL under different soybean treatments. (**A**) Phylum-level composition; (**B**) genus-level composition. SBF, soybean flour-based diet; BSBF, boiled soybean flour-based feed; ESBF, extruded soybean flour-based feed.

Abundance analysis of the three groups at the phylum level revealed that *Proteobacteria*, *Firmicutes*, *Bacteroidota*, and *Actinobacteria* remained the dominant phyla across all three groups. In the SBF group, as previously mentioned, *Firmicutes* (46.46%) and *Proteobacteria* (40.52%) were the dominant phyla, followed by *Bacteroidota* (8.16%) and *Actinobacteria* (4.23%). However, *Firmicutes* (55.56%) exhibited the highest abundance in the BSBF group, followed by *Proteobacteria* (18.76%), *Actinobacteria* (13.04%), and *Bacteroidota* (12.26%). In the ESBF group, *Proteobacteria* (43.55%) had the highest abundance, followed by *Firmicutes* (34.78%), *Bacteroidota* (19.35%), and *Actinobacteria* (2.13%) (Figure 6A, Table S5).

**Figure 7:**
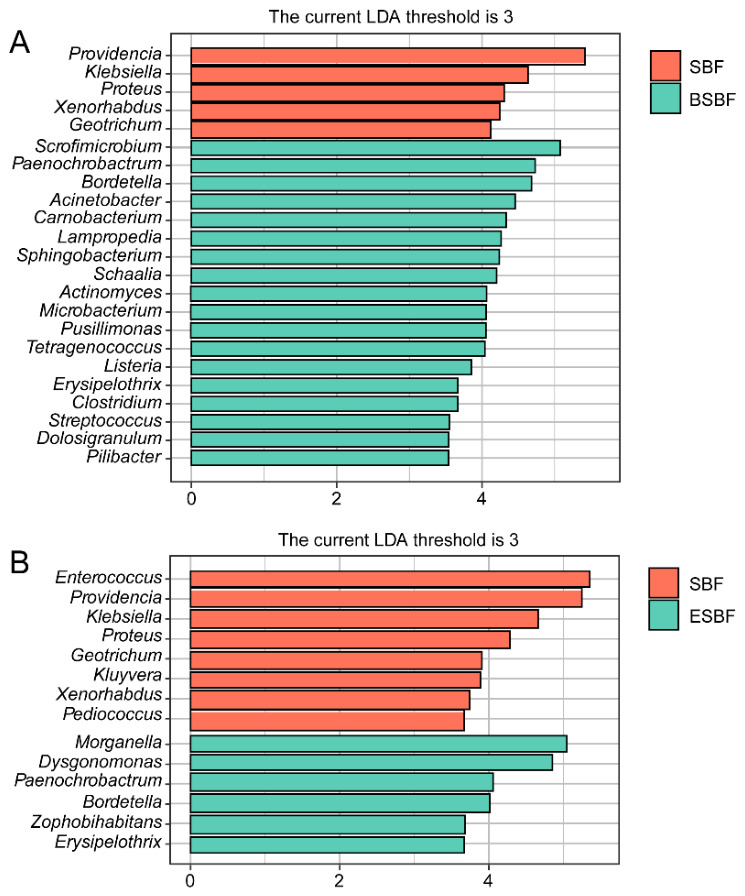
LEfSE analysis of genus-level gut microbiota differences under different soybean treatments. (**A**) BSBF vs. SBF; (**B**) ESBF vs. SBF. SBF, soybean flour-based diet; BSBF, boiled soybean flour-based feed; ESBF, extruded soybean flour-based feed.

The Venn diagram results indicated that there were 754 operational taxonomic units (OTUs) shared among the three groups. The OTUs uniquely present in the SBF, BSBF, and ESBF groups were 52, 333, and 76, respectively (Figure S4A). The circos diagram of taxonomic composition revealed that the top 10 most abundant genera in the three groups were *Enterococcus*, *Providencia*, *Scrofimicrobium*, *Dysgonomonas*, *Vagococcus*, *Morganella*, *Wohlfahrtiimonas*, *Ignatzschineria*, *Alcaligenes* and *Klebsiella* (Figure S4B). Further analysis using a taxonomic composition bar chart showed that the most representative genera in the SBF group were *Enterococcus* (28.42%), *Providencia* (23.76%), *Vagococcus* (15.45%), *Dysgonomonas* (6.08%) and *Klebsiella* (5.03%). The five genera with the highest abundances in the BSBF group were *Enterococcus* (25.56%), *Vagococcus* (17.24%), *Dysgonomonas* (7.79%), *Scrofimicrobium* (7.73%) and *Alcaligenes* (5.21%). In the ESBF group, the top five abundant genera were *Ignatzschineria* (16.37%), *Morganella* (15.71%), *Dysgonomonas* (15.09%), *Enterococcus* (13.09%) and *Vagococcus* (11.79%) (Figure 6B, Table S6). LEfSE analysis (with an LDA threshold of three) was conducted to identify the specificity of taxonomic units with significant differences under different substrate treatment conditions in sample groupings. The results showed that, compared to the untreated SBF group, the abundances of 18 genera, including *Scrofimicrobium*, *Paenochrobactrum*, *Bordetella*, *Acinetobacter* and *Carnobacterium*, increased in the BSBF group (Figure 7A). In contrast, only six genera, namely *Morganella*, *Dysgonomonas*, *Paenochrobactrum*, *Bordetella*, *Zophobihabitans* and *Erysipelothrix*, showed increased abundances in the ESBF group (Figure 7B).

**Figure 8:**
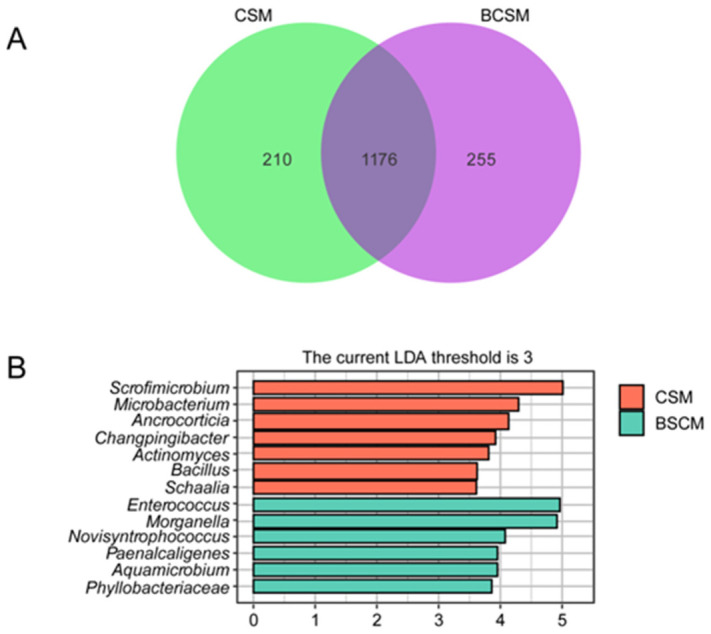
Gut microbiota differences between CSM and BCSM groups. (**A**) Venn diagram of OTUs; (**B**) LEfSE analysis of differential genera. CSM, cottonseed meal-based diet; BCSM, boiled cottonseed meal-based feed.

Given the differences in antinutritional factors between CSM and SBF, we further investigated gut microbiota responses to plant-derived antinutritional factors by comparing untreated (CSM) and boiled (BCSM) cottonseed meal groups. We first evaluated the α-diversity of gut microbiota using Chao1, Shannon, Simpson, Pielou_e and Observed_species indices, and no significant differences were detected between the two groups, indicating that heat treatment did not alter the overall species richness, evenness or diversity of BSFL gut microbiota. (Figure S5A). PCoA-based β-diversity analysis further revealed clear separation of microbial communities along the PCo2 axis, suggesting reshaped community structure despite stable α-diversity (Figure S5B). The circos plot of taxonomic composition illustrated the genus-level distribution pattern of dominant gut bacteria across individual samples from the CSM and BCSM groups, where Enterococcus, Providencia, Scrofimicrobium, and Dysgonomonas were the predominant genera, with distinct abundance proportions between the two treatments (Figure S6). We then analyzed genus-level gut bacterial abundance between the two groups. Venn diagram results showed 1176 shared OTUs, with 210 unique OTUs in the CSM group and 255 unique OTUs in the BCSM group, respectively (Figure 8A). LEfSE analysis (LDA threshold = 3) was used to identify genus-level microbial differences between groups. Higher abundances of *Enterococcus*, *Morganella*, *Novisyntrophococcus*, *Paenalcaligenes*, *Aquamicrobium*, and *Phyllobacteriaceae* were detected in the BCSM group. In contrast, seven genera including *Scrofimicrobium*, *Microbacterium*, *Ancrocorticia*, *Changpingibacter* and *Actinomyces* were enriched in the CSM group (Figure 8B).

### 3.6. Differences in Metabolic Pathways of Intestinal Microbiota Under Different Substrate Treatment Methods

**Figure 9:**
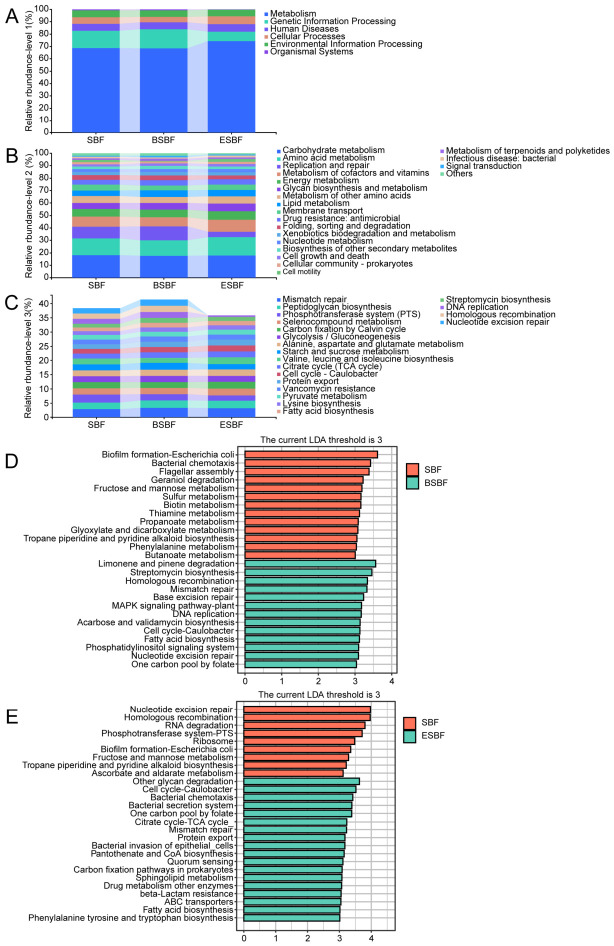
Metabolic pathway analysis under different soybean treatments. (**A**) KEGG level 1; (**B**) KEGG level 2; (**C**) KEGG level 3; (**D**) BSBF vs. SBF; (**E**) ESBF vs. SBF. SBF, soybean flour-based diet; BSBF, boiled soybean flour-based feed; ESBF, extruded soybean flour-based feed.

KEGG analysis was employed to evaluate the metabolic pathways of intestinal microbiota under different substrate treatment conditions, such as extrusion and boiling, for SBF. The results revealed that at level 1, the metabolic pathway with the highest relative abundance was Metabolism, followed by Genetic Information Processing, Human Diseases, Cellular Processes, and Environmental Information Processing (Figure 9A). At level 2, metabolic pathways with relatively high abundances included carbohydrate metabolism, amino acid metabolism, replication and repair, metabolism of cofactors and vitamins, and Energy metabolism (Figure 9B). At the level 3, metabolic pathways with relatively high abundances were Mismatch repair, Peptidoglycan biosynthesis, Phosphotransferase system (PTS), Selenocompound metabolism, and Carbon fixation by Calvin cycle (Figure 9C). LEfSE analysis (with an LDA threshold of three) was conducted to identify differences in level 3 metabolic pathways of gut microbes among the groups with different substrate treatment methods. The results revealed that when comparing the SBF group with the BSBF group, 13 metabolic pathways were upregulated in the SBF group. Among these, four were related to carbohydrate metabolism, and two were associated with the metabolism of cofactors and vitamins. In the BSBF group, 13 metabolic pathways were also upregulated, with five related to replication and repair, and two related to the synthesis of secondary metabolites (Figure 9D). When comparing the SBF group with the ESBF group, nine metabolic pathways were upregulated in the SBF group, of which two were related to carbohydrate metabolism. In the ESBF group, 18 metabolic pathways were upregulated, with two related to lipid metabolism and two related to the metabolism of cofactors and vitamins (Figure 9E).

**Figure 10:**
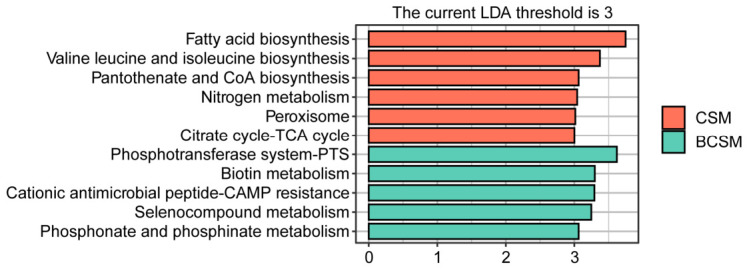
LEfSE analysis of differential metabolic pathways between the CSM and BCSM groups. CSM, cottonseed meal-based diet; BCSM, boiled cottonseed meal-based feed.

Building on the genus-level differences between the CSM and BCSM (Figure 8A), we further explored gut microbial functional changes induced by heat-processed cottonseed meal antinutritional factors using KEGG metabolic pathway analysis. Then, LEfSE analysis (with an LDA threshold of three) was used to assess the functional differences between the two groups. The results indicated that, compared to the BCSM group, the upregulated metabolic pathways in the CSM group were Fatty acid biosynthesis, Valine leucine and isoleucine biosynthesis, Pantothenate and CoA biosynthesis, Nitrogen metabolism, Peroxisome, and Citrate cycle (TCA cycle) (Figure 10).

## 4. Discussion

While BSF’s high bioconversion capacity and nutritional potential are well-documented [5,25], the optimization of plant-based protein feeds for rearing high-quality BSFL remains understudied. Most existing research has focused on organic waste substrates [26,27,28,29], with limited attention to mixed plant-based protein formulations. We incorporated both untreated and antinutritional factor-removed SBF and CSM into experimental feeds, and the elevated protein content of these formulations not only supported favorable larval growth performance but also significantly increased BSFL body protein and fat levels.

Consistent with previous findings that high dietary protein and fat promote larval protein and fatty acid deposition [30], our results showed that BSFL fed high-protein SBF- and CSM-based diets exhibited higher crude protein content, which aligns with reports that BSFL body protein and fat contents vary with feed substrates [31,32,33]. Notably, while SBF and CSM are valuable high-protein feed sources [34], their inherent antinutritional factors are known to reduce nutritional value and potentially impair animal growth [35,36].

Thermal treatment is an effective method for reducing antinutritional factors. Studies have shown that thermal treatment can significantly reduce the activity of trypsin inhibitors in soybeans [37,38], decrease the contents of lectins and β-conglycinin, and improve the digestibility of soybean protein [39]. Additionally, thermal treatment is a common approach for detoxifying and enhancing the nutritional value of CSM [40]. In our study, feeds formulated with SBF or CSM as the main protein sources had higher protein and fat contents compared with the GHDF feed. Untreated SBF and CSM groups both showed inferior growth compared with the GHDF group as follows: the CSM group presented slower development and lower survival, whereas the SBF group suffered more severe growth inhibition. Despite growth retardation, larvae in the SBF and CSM groups accumulated higher body protein and fat contents than the GHDF group, demonstrating that high-protein and high-fat substrates (SBF, CSM) promote protein and fat synthesis in BSFL. Notably, the SBF group exhibited the lowest maximum larval weight, mainly because high levels of soybean-derived antinutritional factors severely inhibited nutrient digestion and absorption, thereby restricting larval growth performance. Thermal-treated groups (BSBF, ESBF, and BCSM groups) showed significantly higher maximum larval weight compared with the untreated SBF and CSM groups, indicating that thermal treatment effectively reduced antinutritional factors in SBF and CSM. Nevertheless, one limitation of the present study is that we did not quantify key antinutritional factors (e.g., trypsin inhibitor activity, gossypol, phytic acid, and lectins) in treated and untreated feeds. Therefore, the exact extent to which thermal processing reduces these compounds remains unconfirmed, which warrants further investigation in future work. These findings provide practical references for producing high-quality BSFL biomass.

The growth of BSFL is influenced not only by the nutritional components of the feed but also by the composition and function of the intestinal microbial community [30]. Research has found that the original nutrients in the feed cannot fully explain the growth and nutrient utilization of the larvae. High-protein or high-fat feeds do not always lead to an increase in the protein and fat proportions in the larvae [30]. The growth of the larvae mainly depends on the interaction between the feed type and the intestinal microbiota. Changing the feed type can alter the core microbial community of the intestinal bacterial community, and the composition and functional potential of the intestinal microorganisms in the larvae affect their growth [30,41,42]. *Firmicutes*, *Proteobacteria*, *Bacteroidetes*, and *Actinobacteria* are the dominant microbial groups in the intestine. *Firmicutes* are widely distributed in soil, the intestine, and other environments, playing crucial roles in various metabolic processes, including cellulose degradation, fatty acid metabolism, and stress resistance enhancement [43,44]. Certain genera within Firmicutes, such as *Ruminococcus* and *Butyrivibrio*, can degrade or neutralize antinutritional factors in plants, such as tannins and polyphenols, demonstrating tolerance to plant-derived antinutritional factors and thus reducing their adverse effects on the host [45]. *Firmicutes* and *Bacteroidetes* are the main producers of short-chain fatty acids. These metabolites not only provide energy for the host but also enhance intestinal barrier function and reduce the direct damage of antinutritional factors to the intestine [44,46,47,48]. *Proteobacteria* are also a group of bacteria with important functions, participating in the nitrogen cycle, sulfur metabolism, and the degradation of organic substances such as proteins and polysaccharides [49]. During the composting process, the abundance of *Proteobacteria* is closely related to the degradation efficiency of organic matter [50,51], indicating their significant role in decomposing complex plant materials. In the process of fermenting kale, fermentation significantly reduced the contents of antinutritional factors such as oxalates and tannins, while also altering the microbial community structure and reducing the proportion of *Proteobacteria* [52], suggesting that the reduction in *Proteobacteria* may be related to the degradation of antinutritional factors. In this study, compared with BSFL fed with the GHFD feed, the relative abundances of *Firmicutes* and *Proteobacteria* in the intestinal microorganisms of the SBF groups significantly increased, and the abundances of *Proteobacteria* and *Bacteroidetes* in the CSM group also increased. These shifts in dominant phyla may contribute to BSFL tolerance to plant antinutritional factors and support gut homeostasis.

Lactic acid is an important organic acid that can lower the intestinal pH, inhibit the growth of pathogenic bacteria (such as *Escherichia shigella*), and increase the abundances of beneficial bacteria (such as *Rikenellaceae*, *Akkermansiaceae*, *Erysipelotrichaceae*, etc.) [53]. Lactic acid can also promote the synthesis of short-chain fatty acids, increase their concentration, and enhance intestinal barrier function [53,54]. In this study, compared with BSFL fed with the GHFD feed or the treated groups (BSBF, ESBF, and BCSM), the abundances of lactic acid- and short-chain fatty acid-producing genera, including *Enterococcus*, *Vagococcus*, *Pediococcus* and *Kluyvera*, were notably higher in the untreated SBF and CSM groups. The enrichment of these functional taxa may help counteract the adverse effects of plant antinutritional factors and maintain intestinal health in BSFL.

To gain a deeper understanding of the role played by the metabolic pathways of intestinal microorganisms in nutrient absorption and resistance to plant antinutritional factors, we analyzed the differences in the metabolic pathways of intestinal microorganisms among each group. In this study, compared with BSFL fed with GHFD or treated SBF or CSM (BSBF, ESBF, and BCSM groups), the upregulated metabolic pathways in the intestinal microorganisms of BSFL fed with untreated SBF or CSM (SBF, CSM groups) were mainly concentrated in carbohydrate-related metabolism, amino acid-related metabolism, cofactor and vitamin-related metabolism, among other pathways. Research has shown that the product of central carbon metabolism, phosphoenolpyruvate, and its by-products can generate a variety of short-chain fatty acids. These substances maintain intestinal barrier integrity, promote the absorption of nutrients by epithelial cells, and prevent the entry of harmful entities and infectious agents [55,56,57]. Intestinal microorganisms also participate in the synthesis of vitamins and essential amino acids, supporting the nutritional needs of the host and facilitating insect development [58]. These findings suggest that the upregulation of these metabolic pathways in our study may contribute to the resistance of BSFL to plant antinutritional factors and nutrient absorption, which is beneficial for the development of BSFL.

## 5. Conclusions

In this study, by using SBF and CSM as the primary protein sources, we developed high-protein/low-fat and high-protein/high-fat formulations for BSFL through treatment and compounding. We successfully cultivated BSFL that exhibited better growth and development compared to those fed with GHFD feed and possessed high protein and fat contents. This demonstrates the feasibility of using high-protein feed formulations to cultivate high-quality BSFL. Meanwhile, the study reveals the significant roles played by *Firmicutes*, *Proteobacteria*, *Bacteroidetes*, as well as lactic acid-producing and short-chain fatty acid-producing bacterial genera in the intestinal microbiota of BSFL in resisting plant antinutritional factors. Additionally, intestinal microbiota-associated metabolic pathways involved in carbohydrates, amino acids, cofactors, and vitamin metabolism contribute to alleviating the adverse effects of plant antinutritional factors and promoting nutrient absorption in BSFL. However, this protective effect was limited and could not completely mitigate the adverse effects of antinutritional factors in untreated SBF and CSM diets.

## Figures and Tables

**Table 1:** Feed ingredients and nutritional analysis.

Feed Ingredient	Crude Protein (CP, %)	Crude Fat (CF, %)	Carbohydrates (Carbs, %)
SBF	35.5	17.3	30.0
CSM	47.0	0.5	36.5
Corn Flour	10.3	3.9	70.5
Wheat Bran	16.2	2.8	63.9
Alfalfa Meal	19.1	2.3	58.0
Rice Bran	14.5	15.5	52.3

Notes: SBF, soybean flour-based diet; CSM, cottonseed meal-based diet.

**Table 2:** Composition of experimental diets.

Group	Feed Ingredients	Dry Weight Ratio (5:3:2)
SBF	Soybean flour + rice bran + corn flour	5:3:2
BSBF	Boiled soybean flour + rice bran + corn flour	5:3:2
ESBF	Extruded soybean flour + rice bran + corn flour	5:3:2
CSM	Cottonseed meal + rice bran + corn flour	5:3:2
BCSM	Boiled cottonseed meal + rice bran + corn flour	5:3:2
GHFD	Wheat bran + alfalfa meal+ corn meal	5:3:2

Notes: SBF, soybean flour-based diet; CSM, cottonseed meal-based diet; BSBF, boiled soybean flour-based feed; ESBF, extruded soybean flour-based feed; BCSM, boiled cottonseed meal-based feed; GHDF, Gainesville House Fly Diet.

**Table 3:** Nutritional profiles of each experimental diet.

Experimental Group	Crude Protein (CP, %)	Crude Fat (CF, %)	Carbohydrates (Carbs, %)
SBF/BSBF/ESBF	24.16	14.08	44.79
CSM/BCSM	29.91	5.68	48.04
GHDF	15.89	2.87	63.45

Notes: SBF, soybean flour-based diet; CSM, cottonseed meal-based diet; BSBF, boiled soybean flour-based feed; ESBF, extruded soybean flour-based feed; BCSM, boiled cottonseed meal-based feed; GHDF, Gainesville House Fly Diet.

**Table 4:** Nutritional analysis of BSFL fed different diets.

Groups	Crude Protein (CPs, %)	Crude Fat (CF, %)
SBF	49.95 ± 1.05 bc	26.19 ± 0.20 c
BSBF	55.12 ± 0.18 a	27.25 ± 0.33 c
ESBF	41.63 ± 0.29 c	29.45 ± 0.55 bc
CSM	53.05 ± 0.33 b	32.77 ± 0.28 b
BCSM	59.88 ± 0.59 a	35.82 ± 0.38 a
GHDF	46.60 ± 0.19 c	22.67 ± 0.33 d

Note: SBF, soybean flour-based diet; CSM, cottonseed meal-based diet; BSBF, boiled soybean flour-based feed; ESBF, extruded soybean flour-based feed; BCSM: boiled cottonseed meal-based feed; GHDF, Gainesville House Fly Diet. Different lowercase letters in the same column indicate significant differences among treatments (*p* < 0.05). The same letter indicates no significant difference (*p* ≥ 0.05).

## Data Availability

The original contributions presented in this study are included in the article/Supplementary Material. Further inquiries can be directed to the corresponding authors.

## Supplementary Materials

The following supporting information can be downloaded at https://www.mdpi.com/article/10.3390/insects17070675/s1. Figure S1. Gut microbiota sequencing quality and diversity analysis in BSFL fed different diets. (A) Alpha-diversity indices; (B) PCoA plot of β diversity. SBF, soybean flour-based diet; CSM, cottonseed meal-based diet; GHDF, Gainesville House Fly Diet. Figure S2. Genus-level microbial distribution in BSFL fed different diets. (A) Venn diagram of OUT; (B) Circos plot of top 10 genera. SBF, soybean flour-based diet; CSM, cottonseed meal-based diet; GHDF, Gainesville House Fly Diet. Figure S3. Gut microbiota diversity in BSFL fed SBF with different treatments. (A) Alpha-diversity indices; (B) PCoA plot of β diversity. SBF, soybean flour-based diet; BSBF, boiled soybean flour-based feed; ESBF, extruded soybean flour-based feed. Figure S4. Genus-level microbial distribution in BSFL under different soybean treatments. (A): Venn diagram of OTUs; (B) Circos plot of 10 genera. SBF, soybean flour-based diet; BSBF, boiled soybean flour-based feed; ESBF, extruded soybean flour-based feed. Figure S5. Gut microbiota diversity in BSFL fed CSM with different treatments. (A) Alpha-diversity indices; (B) PCoA plot of β-diversity. CSM, cottonseed meal-based diet; BCSM, boiled cottonseed meal-based diet. Figure S6. Circos plot of genus-level gut microbiota composition in BSFL fed CSM and BCSM diets. CSM, cottonseed meal-based diet; BCSM, boiled cottonseed meal-based diet. Table S1. Growth and developmental characteristics. Table S2. Quality control parameters for the metagenome analyses. Table S3. Taxonomic composition analysis (phylum level) in BSFL Fed with Different Diets. Table S4. Taxonomic composition analysis (genus level) in BSFL Fed with Different Diets. Table S4. Taxonomic composition analysis (genus level) in BSFL Fed with Different Diets. Table S5. Taxonomic composition analysis (phylum level) in BSFL under different substrate treatment methods. Table S6. Taxonomic composition analysis (genus level) in BSFL under different substrate treatment methods.

## Abbreviations

The following abbreviations are used in this manuscript:

BSF	Black soldier fly
BSFL	Black soldier fly larvae
SBF	Soybean flour
CSM	Cottonseed meal
GHFD	Gainesville House Fly Diet
ESBF	Extruded soybean flour
BSBF	Boiled soybean flour
