# Supplementary material for: Effects of Soybean- and Cottonseed-Based Diets on Growth Performance and Gut Microbiota of Black Soldier Fly Larvae
Source: Insects. 2026 Jun 28;17(7):675. doi: 10.3390/insects17070675 (PMC13409921; doi:10.3390/insects17070675)
Supplement: Supplementary file 1 [file insects-17-00675-s001.zip › Supplementary data figures.pdf]

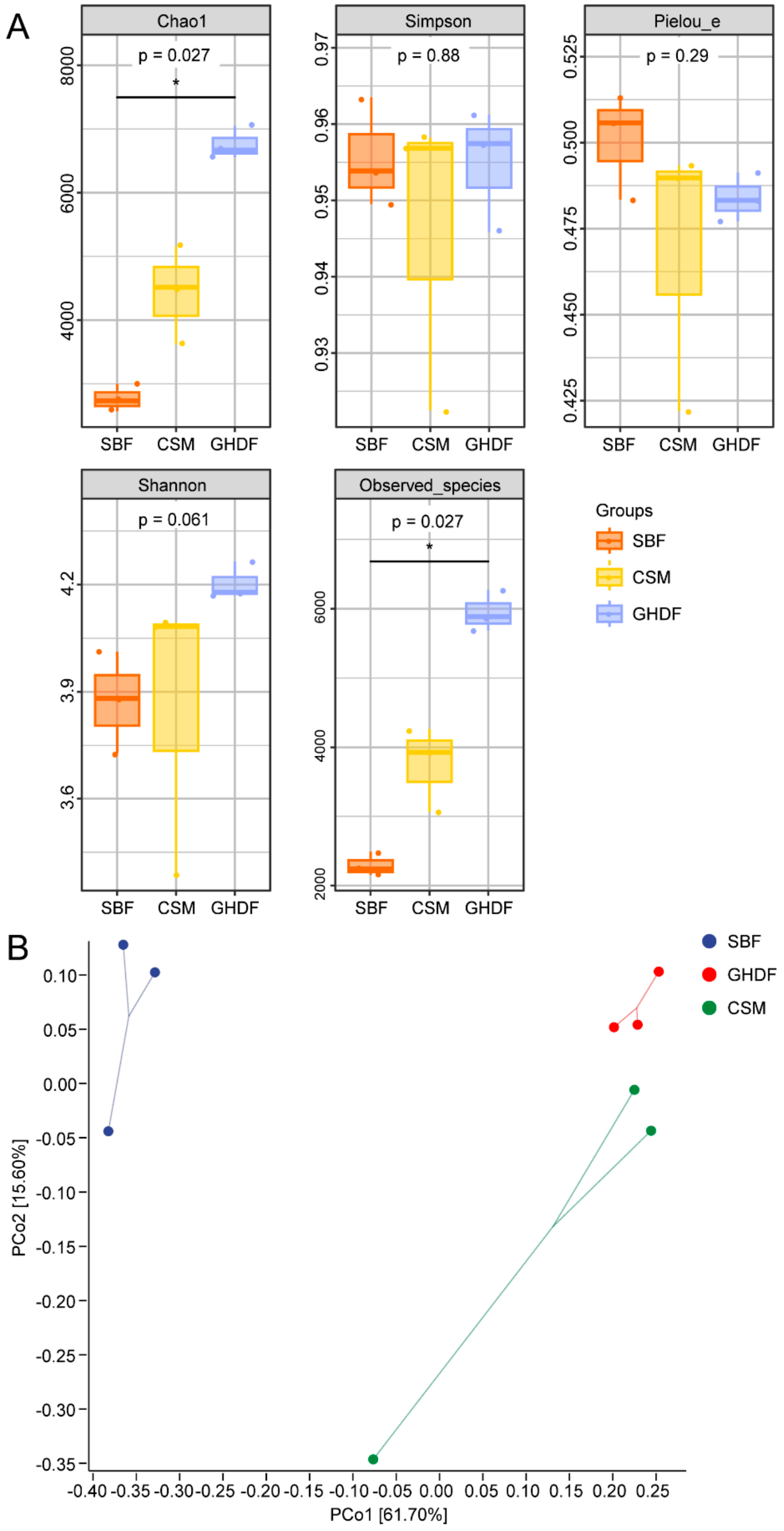

Supplementary data figures

Figure S1. Gut microbiota sequencing quality and diversity analysis in BSFL fed different diets. (A) Alpha-diversity indices; (B) PCoA plot of β-diversity. SBF, soybean flour-based diet; CSM, cottonseed meal-based diet; GHDF, Gainesville House Fly Diet.

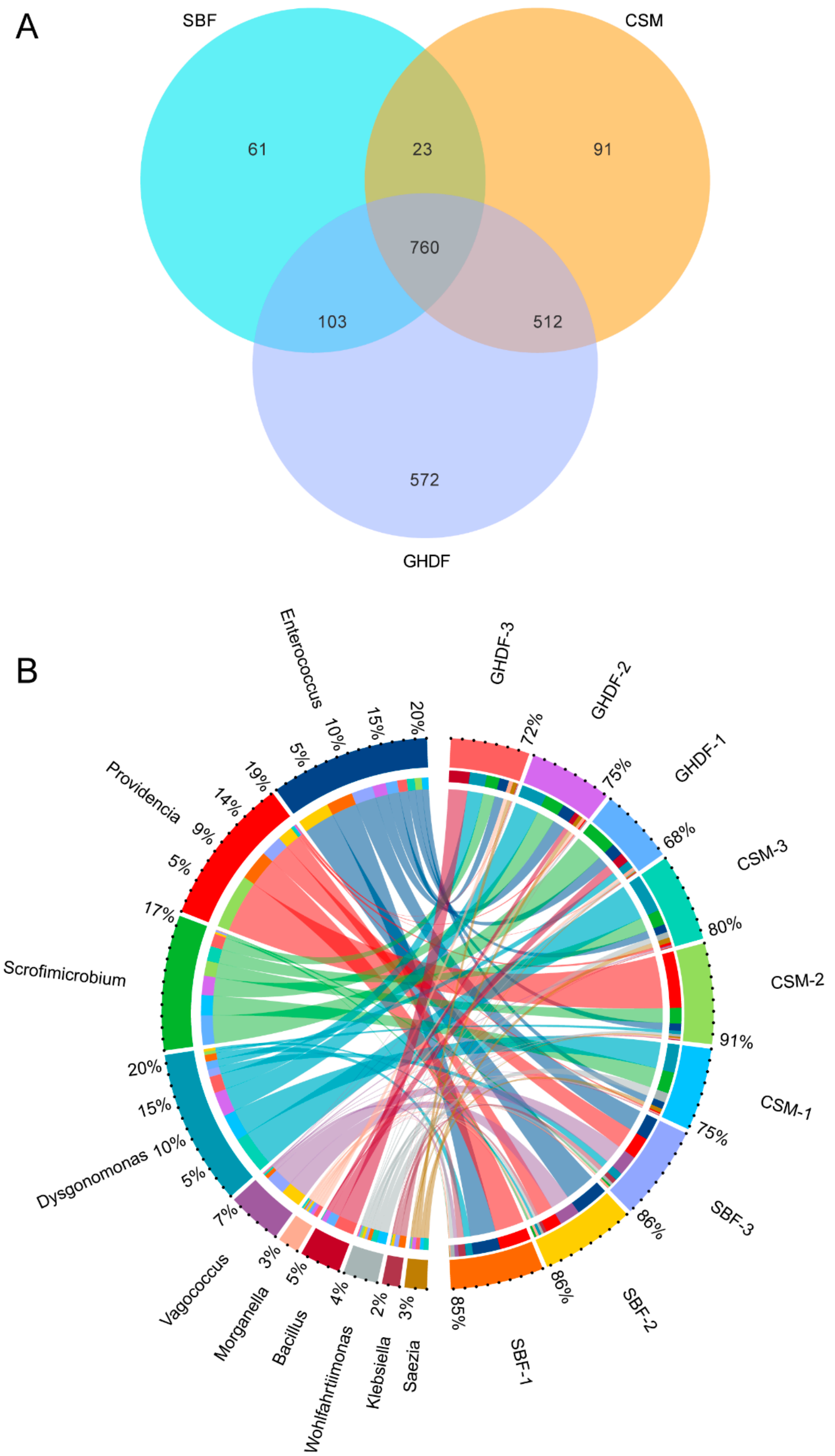

Figure S2. Genus-level microbial distribution in BSFL fed different diets. (A) Venn diagram of OUT; (B) Circos plot of top 10 genera. SBF, soybean flour-based diet; CSM, cottonseed meal-based diet; GHDF, Gainesville House Fly Diet.

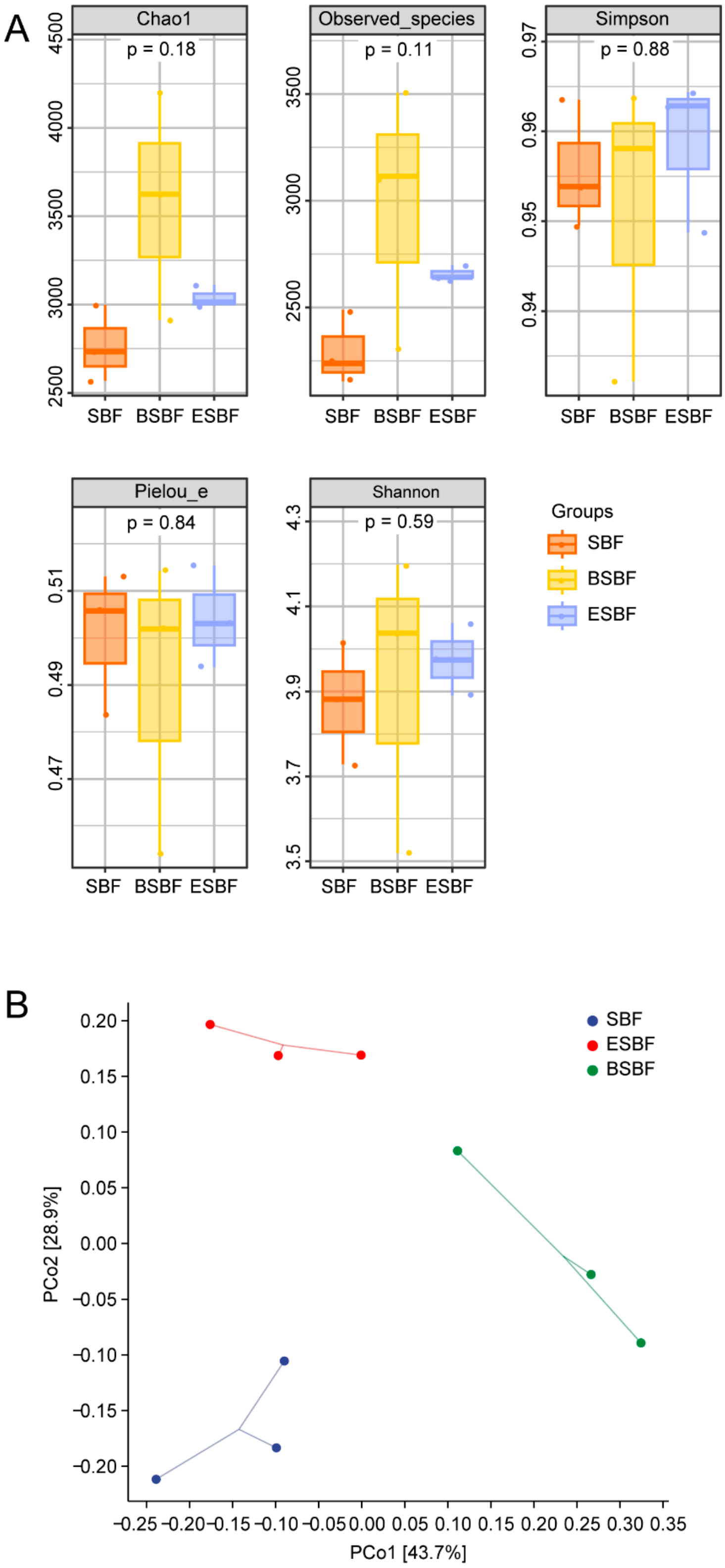

Figure S3. Gut microbiota diversity in BSFL fed SBF with different treatments. (A) Alpha-diversity indices; (B) PCoA plot of β-diversity. SBF, soybean flour-based diet; BSBF, boiled soybean flour-based feed; ESBF, extruded soybean flour-based feed.

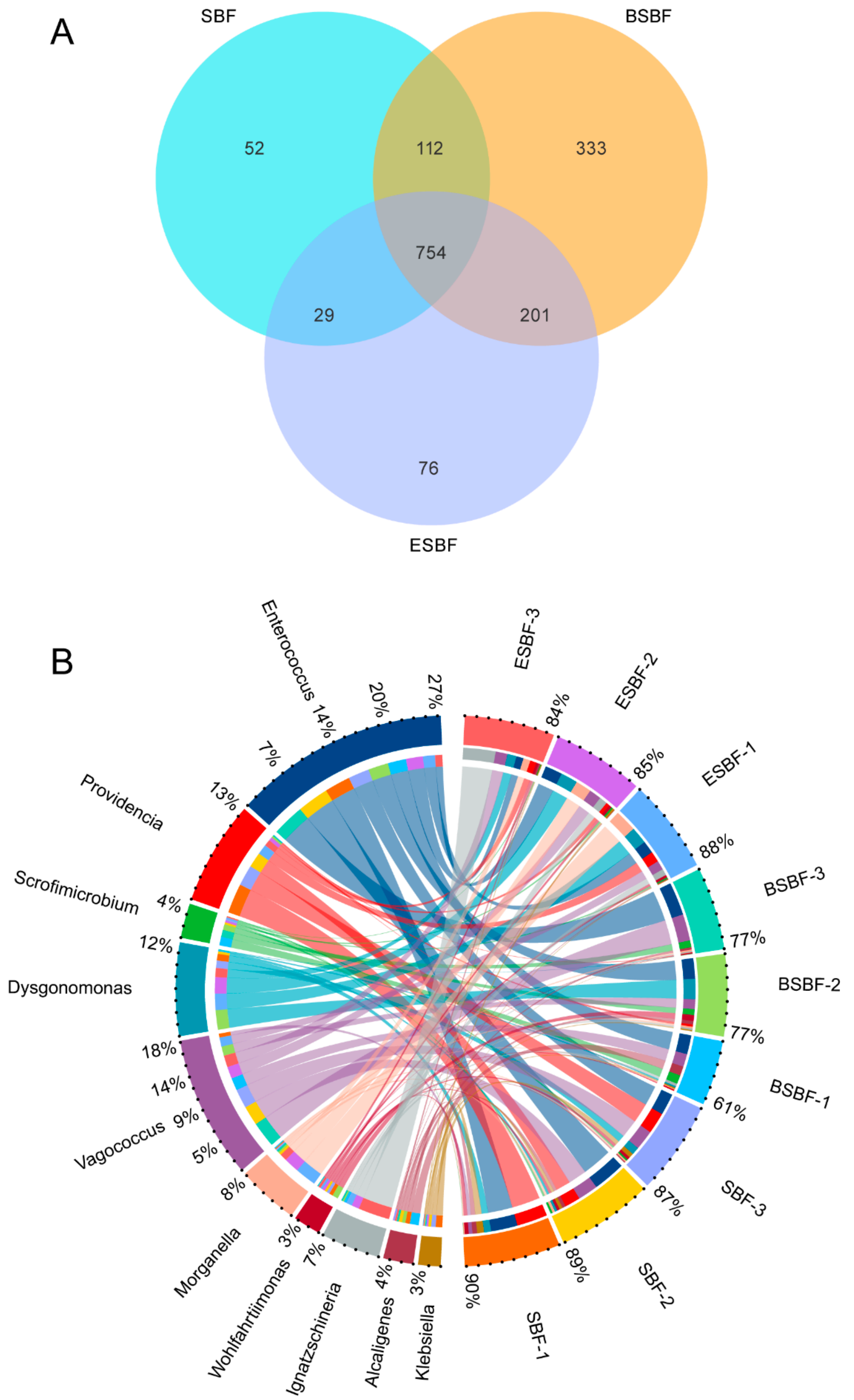

Fig S4. Genus-level microbial distribution in BSFL under different soybean treatments. (A): Venn diagram of OTUs; (B) Circos plot of 10 genera. SBF, soybean flour-based diet; BSBF, boiled soybean flour-based feed; ESBF, extruded soybean flour-based feed.

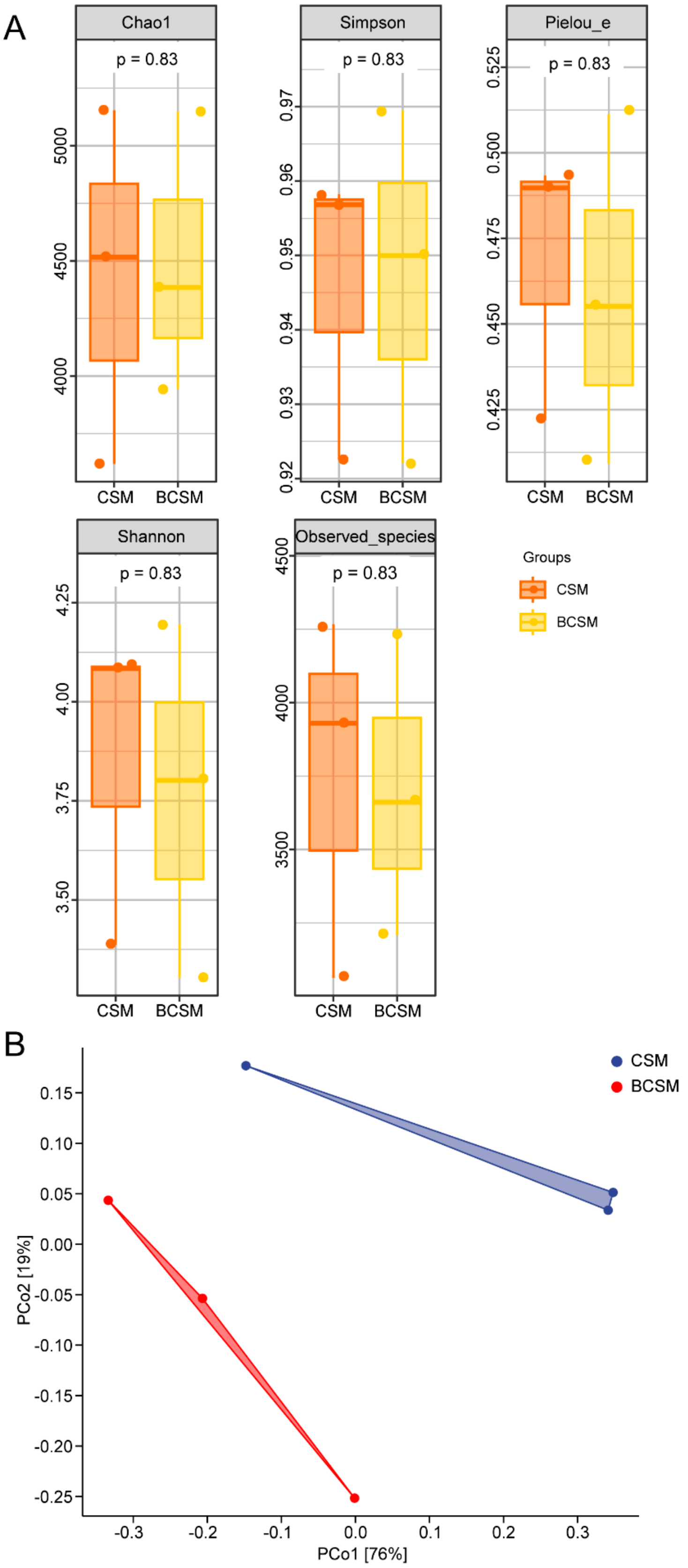

Figure S5. Gut microbiota diversity in BSFL fed CSM with different treatments. (A) Alpha-diversity indices; (B) PCoA plot of β-diversity. CSM, cottonseed meal-based diet; BCSM, boiled cottonseed meal-based diet.

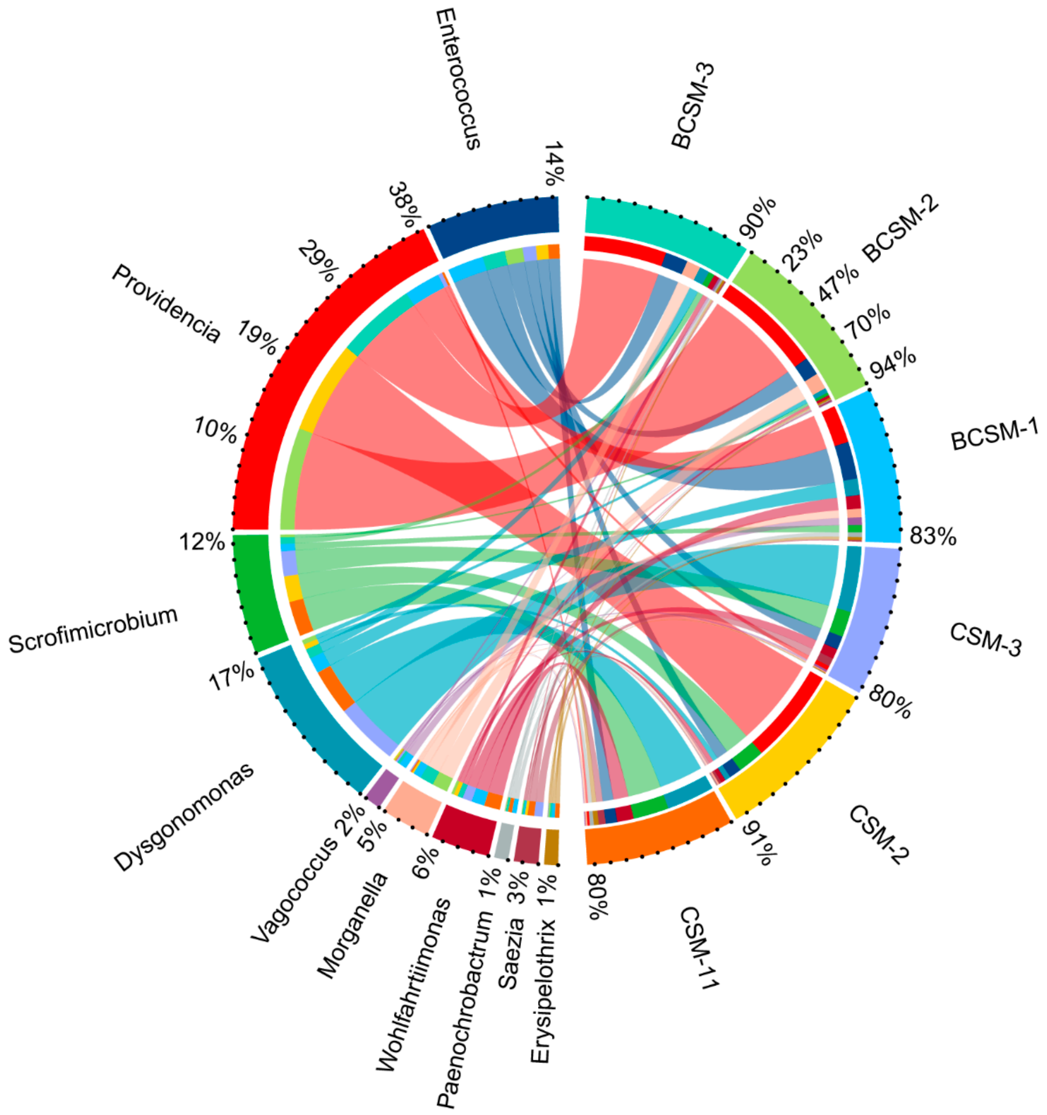

Figure S6. Circos plot of genus-level gut microbiota composition in BSFL fed CSM and BCSM diets. CSM, cottonseed meal-based diet; BCSM, boiled cottonseed meal-based diet.
